# Supplementary material for: The automatic parameter-exploration with a machine-learning-like approach: Powering the evolutionary modeling on the origin of life
Source: PLoS Comput Biol. 2021 Dec 29;17(12):e1009761. doi: 10.1371/journal.pcbi.1009761 (PMC8752021; doi:10.1371/journal.pcbi.1009761)
Supplement: S1 Table — (PDF) [file pcbi.1009761.s001.pdf]

Table S1. Parameters used in the automatic exploration

Parameters	Descriptions
PNF	Probability of nucleotide formation (not catalyzed by NSR)
PNFR	Probability of nucleotide formation under the catalysis of NSR
PND	Probability of nucleotide decay
PRL	Probability of the random ligation of nucleotides and
PBB	Probability of breaking a phosphodiester bond
PAT	Probability of attracting a substrate by a template
PFP	Probability of the false base-pairing
PMV	Probability of the movement of raw material to an adjacent grid

* For a detailed explanation of these parameters, as well as that of the whole model (including the other five parameters which are not involved in the parameter-exploration here), please see the original paper [20].
