# Supplementary figures and images for: The automatic parameter-exploration with a machine-learning-like approach: Powering the evolutionary modeling on the origin of life
Source: PLoS Comput Biol. 2021 Dec 29;17(12):e1009761. doi: 10.1371/journal.pcbi.1009761 (PMC8752021; doi:10.1371/journal.pcbi.1009761)

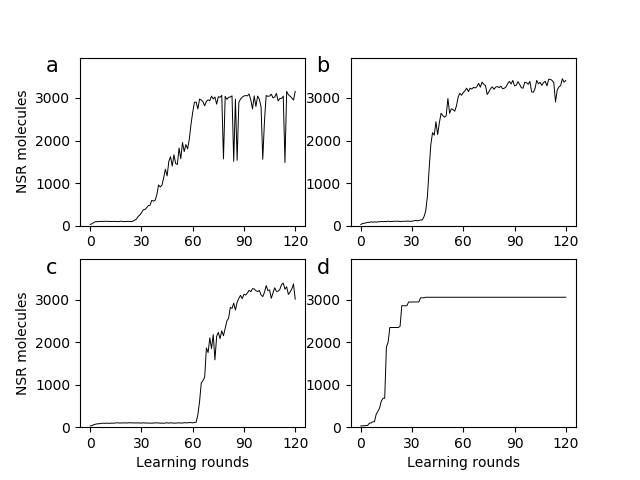

Supplement: S1 Fig — The starting value of parameters are the same as those in Fig 2A: i.e., PNF = 4×10^−3^, PNFR = 0.02, PND = 1×10^−3^, PRL = 2×10^−5^, PBB = 1×10^−5^, PAT = 0.5, PFP = 0.1, and PMV = 1×10^−3^. The learning rate e = 0.5 (corresponding to the blue line in Fig 2A). As with the cases in Fig 2, the objective function is the number of NSR molecules at step 1.5×10^5^ in the evolutionary dynamics (refer to Fig 1B). (a) When a change of one parameter does not bring about the value change of the objective function, in the next round of learning it is adjusted downwards instead of upwards. (b) The change of a parameter concerning the learning rate is implemented by the rule of multiplication, instead of the rule of addition. (c) Adoption of the implementation strategy in b with the adjusting strategy of a. (d) Here, instead of the gradient ascent, the approach of coordinate ascent is used. See Methods for a detailed description about all these variations. (TIF) [file pcbi.1009761.s002.tif]

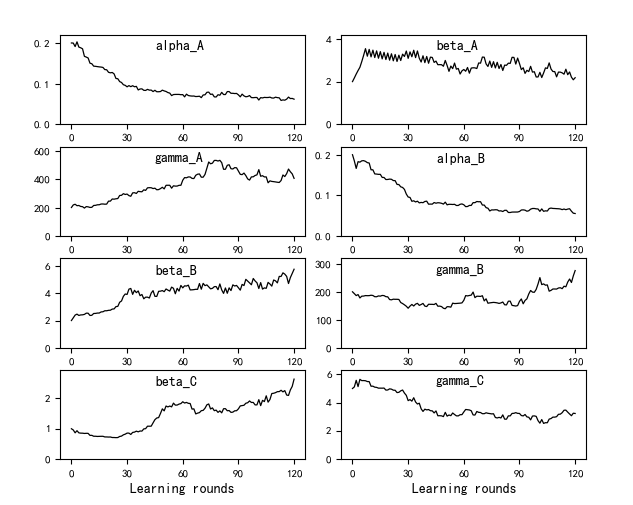

Supplement: S2 Fig — The improvement of replicator length of this case is shown in Fig 8A. The vertical axis of a subfigure represents the value of the corresponding parameter whose name is shown within the panel. The eight parameters are adjusted simultaneously in a learning round. (TIF) [file pcbi.1009761.s003.tif]

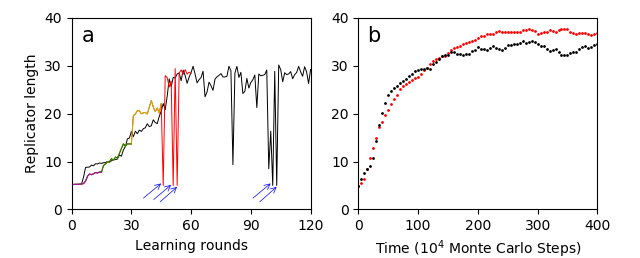

Supplement: S3 Fig — (a) The black line is the same as that in Fig 8A, whose objective function, all through the learning process, is the average length of replicators at step 100×104. The learning process denoted by the magenta segment (from round 1 to 15) uses an objective function of the replicator length at step 10×104, with a starting point of parameter setting the same as that of the black line case (refer to Fig 7B); the learning process denoted by the green segment (from round 16 to 30) uses an objective function of the replicator length at step 20×104, with a starting point of parameter setting achieved from the previous learning process (the magenta segment); the brown segment (from round 31 to 45) uses an objective function of the replicator length at step 40×104, with a starting point of parameter setting achieved from the previous learning process (the green segment); the red segment (from round 46 to 60) uses an objective function of replicator length at step 100×104, with a starting point of parameter setting achieved from the previous learning process (the brown segment). The arrows indicate “odd points” (see the legend of Fig 8 for an explanation). (b) The black dots represent the improved evolutionary dynamics (in comparison with Fig 7B) by adopting the parameter set at the 60th learning round of the black line case in a, which does not use the progressive strategy (actually the same as shown in Fig 8B), while the red dots represent the improved evolutionary dynamics (in comparison with Fig 7B) by adopting the parameter set at the 60th learning round of the red line case in a, which uses the progressive strategy. (TIF) [file pcbi.1009761.s004.tif]
