# Supplementary material for: The automatic parameter-exploration with a machine-learning-like approach: Powering the evolutionary modeling on the origin of life
Source: PLoS Comput Biol. 2021 Dec 29;17(12):e1009761. doi: 10.1371/journal.pcbi.1009761 (PMC8752021; doi:10.1371/journal.pcbi.1009761)
Supplement: Attachment — Submitted filename: response-to-reviewers.docx [file pcbi.1009761.s005.docx]

We thank the reviewers for their comments on our paper. The following is our response to their critiques and suggestions.

**Reviewer #1:**

The paper starts from the important point that many models used in evolutionary biology and origin of life studies have many parameters, and that finding suitable values of these parameters is difficult by trial and error. The paper gives a method of automatic learning of parameters which directs the parameters via a gradient ascent method towards optimal values. The model of nucleotide synthetase replication is taken from the author's previous work. This is an interesting model related to the RNA World. The current paper demonstrates that good parameter sets for this model can be found by the automatic learning method. I think this is a useful and interesting paper. In practice, the method requires evaluating the model on many different parameter sets. The practicality of the method will depend on how fast the model can be run on each parameter set.

--- Yes, the reviewer’s description on our work is quite accurate, thanks.

**Philosophical points:**

We always have a tendency to want to "prove that our model is right" by finding parameters where our model produces a good outcome in comparison to somebody else's model. If authors select parameters by hand, they could be accused of "cherry picking" parameters that demonstrate a preconceived desired outcome. It seems that this automated method does not get rid of this problem, because there will always be parameters in the learning algorithm itself that can be chosen – such as the outcome function O which is optimized, and details of the rules by which the parameters are adjusted. My point is simply that the automated method does not removed potential human biases.

---Indeed, as the reviewer noted, for theoretical modeling, this problem often exists. However, the focus of our work here is not on this problem, but to improve the efficiency of choosing parameter values. As mentioned in the first paragraph of Discussion, the evolution during the origin of life is remarkably characterized by the tendency from simplicity to complexity, which tends to be an unusual phenomenon in nature. Therefore, any relevant hypothetic scene in this area (suggesting a simplicity-to-complexity evolution), if only supported by modeling with certain parameter-set(s), deserves our attention. In fact, what we care the most here is whether the result of the parameter-exploration is an artifact – that is, for any hypothetic scene, there might be always some parameter sets supporting it – if so, the modeling should be problematic. In the case involved in the present study, we have test such a concern for the possibility of the spread of an NSR losing its function, and the result showed that no parameter sets can be learned out for the spread of the “non-functional” NSR (Fig 5b).

It is often interesting to present results of a model as a function of changing values of a particular parameter. In this way we can see that good outcomes of a model depend on certain values of the parameter. In other words, it is useful see cases where the model does not work as well as the optimum parameter values.

---Yes, it is certainly useful to see cases where the model does not work as well as the optimum parameter values. In fact, if we have obtained the optimum parameter set, it is easy to test the influence of different parameters by altering them separately. The detailed information on the optimal parameter-setting and the results of the subsequent testing by separate parameter adjustments would provide clues for us to judge the likelihood of such a scene in history based on our extant knowledge concerning prebiotic chemistry and environments, or for such a judgement in future if by now we have not got relevant knowledge. That is just where the significance of our “reverse” computer modeling lies. **A corresponding explanation has been added into Discussion (Line 266-273 – in the clean version, the same below).** Thanks.

**Technical points:**

The parameter e is called the learning rate. But e is used in two different ways in the method. Firstly a parameter is changed from p to p(1+e). The change is Deltap = pe, and the gradient is DeltaO/Deltap. Secondly, the parameters are adjusted by an amount proportional to e (equation 5). In the second sense, e is indeed a learning rate because it determines the rate of change of parameters, but in the first sense, e is not a learning rate. It simply determines the small change Deltap used to estimate the gradient. It is therefore slightly confusing to call e the learning rate on line 336. There is no particular reason why the e used for Deltap has to be the same as the e used for adjusting the parameters.

---Yes, this is an important issue of conception, thanks. Indeed, *e* in the first sense simply defines the small change of the parameters used to estimate the gradient. For a deterministic model, in which the gradient could be determined by analytic methods, the usage of *e* in this sense is often unnecessary. But for the Monte-Carlo model as demonstrated here, we need a “testing rate” to estimate the gradient. Though in principle, the “testing rate” and the true “learning rate” need not be the same, they should adopt a similar scale in practice. For instance, when we want to conduct a fast learning and thus use a great “learning rate” for adjusting the parameters, then using a very small “testing rate” for an accurate estimation of the gradient (which would bring great computational cost) would appear unnecessary; on the other hand, if we would like to conduct a smooth learning and thus use a small learning rate, then using a relatively great “testing rate” would tend to be insufficient to estimate a gradient accurate enough for a valid learning step forward. Therefore, for simplification in practice, here we adopt the same value and use a unitary representation for the two rates. **We have added an explanation for this point in Methods (Line 409-423)**.

In a deterministic model we would expect that the estimated gradient DeltaO/Deltap has a well-defined limit dO/dp when e tends to 0, and that the estimated gradient should not depend much on *e*. In a stochastic model, the outcome will fluctuate, even if the model is run twice with the same parameters. So the gradient will be dominated by statistical error if e is too small. Did the authors ever observe this problem in their simulations? For the results shown in Fig 2, the small e case seems to give quite smooth results, so probably e was not too small.

--- Yes, as explained above, for a deterministic model, the estimation of gradient may be unnecessary. Indeed, for a stochastic model, in principle, the outcome will fluctuate. But for the Monte-Carlo simulation on an evolutionary process concerning the origin of life, the probabilities involved in the model are usually sampled numerous times over a large quantity of time steps (e.g., see the two cases mentioned in this paper), and thus the fluctuation would not matter if the testing rate is not too small. Certainly, if the testing rate is approaching zero and the scale of time steps for the Monte-Carlo simulation is not too long, it would be easy to observe this problem in our simulation mentioned by the reviewer.

On the other hand, the large e case appears to be unstable, with big drops in the O function occurring frequently. I would suggest that the reason for this is that the parameters are adjusted by an amount that depends on DeltaO/Max-abs-DeltaO (in equation 5). If the parameters are approaching an optimum, then Max-abs-DeltaO will be small, and all the parameters will be shifted by a big amount, thus moving the model a long way from the optimum. It would probably be better to shift the parameters by an amount proportional simply to DeltaO. Then the algorithm would smoothly approach the optimum.

---No, perhaps this is a misunderstanding. Max-abs-DeltaO represents the biggest one among the absolute values of those DeltaOs. That is, all DeltaOs would be smaller in scale than Max-abs-DeltaO, and thus DeltaO/Max-abs-DeltaO is a value between -1 and 1. Therefore, the unstability should not come from the reason mentioned by the reviewer. In fact, the unstability should be similar to the common situation for any optimization when the optimum is approaching. An ordinary solution to this problem in machine-learning is to adjust the learning rate progressively, that is, e would decrease gradually when the optimum is approaching. But here such a strategy (or some strategy alike) is not necessary because the aim of parameter-exploration for the evolutionary modeling studies on the origin of life is not to seek an optimum in a pure sense, but to know roughly about the existence of a good parameter set favoring a hypothetic scene. Certainly, for any potential goals to seek an absolute optimum in future work of this area, improvements along this line would be readily to achieve – simply based on those relevant techniques developed in the field of machine-learning [40]. **We have added this explanation into Discussion (Line 274-284)**.

Another option would be to make small random changes to the parameters, and then accept all moves that increase O and accept moves that decrease O with a smaller probability that depends on DeltaO. This would be a variant of the Metropolis algorithm. This would have the potential advantage of avoiding being trapped permanently in local optima, and also it does not require the calculation of the gradient, which is time-consuming because the model has to be evaluated separately for changes in each parameter.

---Yes, for the automatic parameter-exploration, there may be other learning algorithms, as also suggested by Reviewer 2. Here the reviewer talked about an interesting idea. It does not require the calculation of the gradient, and accepting the decrease of the objective function to an extant might avoid being trapped permanently in local optima. But the Metropolis algorithm is sometime not quite efficient in finding an optimum. Concerning the automatic parameter-exploration, perhaps further investigation on this method is needed to draw a conclusion. **Corresponding descriptions and comments on this idea have been added into Discussion (Line 325-333)**. Many thanks

**Reviewer #2:**

This is a valuable contribution, and may help move the field further. There is only one aspect that I am missing from the discussion. The Authors apply mostly the gradient ascent algorithm, but there are several others. Do they have a comment what they would expect (or not) from alternatives? Evolutionary algorithms naturally come to mind in this context. At least some of the investigated parameters could be made genotypic properties and thus evolvable. Parameters that are simply given by chemistry in the given environment (such as probability of breaking a phosphodiester bond) cannot be treated in this way, but others can. I think this would be interesting. It would be a bit similar to population genetic models that have evolvable mutation or recombination rates.

---Thanks. Yes, applying evolutionary algorithms in the automatic parameter-exploration is also a good idea (Reviewer 1 has suggested the Metropolis algorithm). For instance, we may treat some investigated parameters as genotypes and the objective function as the phenotype. Genotypes favoring the improvement of the phenotype is selected iteratively over many rounds of “reproduction” where mutation and recombination of the genotypes are introduced. Then, starting from a “bad” parameter-set, perhaps a good parameter-set favoring the hypothetic scene may gradually emerge in the “parameters’ evolution”. Doubtlessly, we could expect an interesting study in future to evaluate the effectiveness and the efficiency (if effective) of the evolutionary algorithms in regard to the automatic parameter-exploration concerning the modeling work on the origin of life. **Corresponding descriptions and comments on this idea have been added into Discussion (Line 334-341).** Many thanks.
